# Nox-2-Mediated Phenotype Loss of Hippocampal Parvalbumin Interneurons Might Contribute to Postoperative Cognitive Decline in Aging Mice

**DOI:** 10.3389/fnagi.2016.00234

**Published:** 2016-10-13

**Authors:** Li-Li Qiu, Dan Luo, Hui Zhang, Yun S. Shi, Yan-Jun Li, Dan Wu, Jiang Chen, Mu-Huo Ji, Jian-Jun Yang

**Affiliations:** ^1^Zhongda Hospital, School of Medicine, Southeast UniversityNanjing, China; ^2^Department of Anesthesiology, Jinling Hospital, School of Medicine, Nanjing UniversityNanjing, China; ^3^MOE Key Laboratory of Model Animal for Disease Study, Model Animal Research Center, Nanjing Biomedical Research Institute, Nanjing UniversityNanjing, China

**Keywords:** postoperative cognitive decline, parvalbumin, Nox2, aging, hippocampus, apocynin

## Abstract

Postoperative cognitive decline (POCD) is a common complication following anesthesia and surgery, especially in elderly patients; however, the precise mechanisms of POCD remain unclear. Here, we investigated whether nicotinamide adenine dinucleotide phosphate (NADPH) oxidase mediated-abnormalities in parvalbumin (PV) interneurons play an important role in the pathophysiology of POCD. The animal model was established using isoflurane anesthesia and exploratory laparotomy in 16-month-old male C57BL/6 mice. For interventional experiments, mice were chronically treated with the NADPH oxidase inhibitor apocynin (APO). Open field and fear conditioning behavioral tests were performed on day 6 and 7 post-surgery, respectively. In a separate experiment, brain tissue was harvested and subjected to biochemical analysis. Primary hippocampal neurons challenged with lipopolysaccharide (LPS) *in vitro* were used to investigate the mechanisms underlying the oxidative stress-induced abnormalities in PV interneurons. Our results showed that anesthesia and surgery induced significant hippocampus-dependent memory impairment, which was accompanied by PV interneuron phenotype loss and increased expression of interleukin-1β (IL-1β), markers of oxidative stress and NADPH oxidase 2 (Nox2) in the hippocampus. In addition, LPS exposure increased Nox2 level and decreased the expression of PV and the number of excitatory synapses onto PV interneurons in the primary hippocampal neurons. Notably, treatment with APO reversed these abnormalities. Our study suggests that Nox2-derived reactive oxygen species (ROS) production triggers, at least in part, anesthesia- and surgery-induced hippocampal PV interneuron phenotype loss and consequent cognitive impairment in aging mice.

## Introduction

Postoperative cognitive decline (POCD) is a recognized clinical phenomenon that refers to cognitive impairment occurring in patients after anesthesia and surgery, especially in the elderly (Terrando et al., 2011). One multicenter trial demonstrated that in patients older than 60 years who were recovering from major non-cardiac surgery, POCD was present in 25.8% of patients at 1 week and 9.9% at 3 months postoperatively (Moller et al., 1998). Thus, POCD has drawn significant attention from the public and from the scientific community because of its clinical impact in terms of negative outcomes and financial burden on the health care system (Monk et al., 2008; Steinmetz et al., 2009). The cause of POCD is thought to be multifactorial and may involve a combination of patient, surgical and anesthetic factors (Monk et al., 2008; Steinmetz et al., 2009; Terrando et al., 2011; Hovens et al., 2012). Several potential mechanisms, including oxidative stress (An et al., 2013; Yuan et al., 2014) neuroinflammation (Cibelli et al., 2010; Zhang et al., 2014), alterations in neurotransmitters (Fan et al., 2014) and β-amyloid accumulation (Xu et al., 2014) have been proposed to underlie the pathogenesis of POCD. Yet, the mechanisms by which these pathological events lead to cognitive impairment remain to be elucidated.

The cerebral cortex contains both excitatory glutamatergic neurons and inhibitory GABAergic interneurons that work together to maintain the balance between inhibition and excitation, which is necessary for the normal functioning of the cortex (Ouellet and de Villers-Sidani, 2014). The inhibitory GABAergic interneurons constitute 10–20% of all cortical neurons, of which 40–50% are parvalbumin (PV) interneurons that regulate the activity of neural networks through perisomatic inhibition onto the pyramidal cells (Lewis et al., 2005; Behrens et al., 2007; Donato et al., 2013). PV interneurons are vital for the generation of gamma oscillations (Bartos et al., 2007; Sohal et al., 2009), which provides a temporal structure for information processing including attention, perception and working memory (Fuchs et al., 2007; Gulyás et al., 2010; Lewis et al., 2012). However, the dysfunction or phenotype loss of PV interneurons (represented by a decrease in the expression of GABA-related genes, such as the 67-kDa isoform of glutamate decarboxylase (GAD 67) and PV) has been closely linked to the cognitive impairment that is associated with many major neuropsychiatric disorders including Alzheimer’s disease (Verret et al., 2012), schizophrenia (Fazzari et al., 2010; Yang et al., 2013), autism (Orekhova et al., 2007) and epilepsy (Ellender et al., 2014; Shiri et al., 2015). Although the precise mechanism remains unknown, oxidative stress is considered to be a vital factor that contributes to PV interneuron phenotype loss because of the high metabolic requirements (Steullet et al., 2010; Cabungcal et al., 2013).

Although mitochondria are considered to be the major source of intracellular reactive oxygen species (ROS), several lines of evidence suggest that various subunits of nicotinamide adenine dinucleotide phosphate (NADPH) oxidase in the plasma membrane are highly expressed in cortical neurons and can also generate ROS. NADPH oxidase, a multi-unit enzyme, is composed of several subunits including Nox1-5 and Dual oxidase 1 (Duox 1) and 2 (Bedard and Krause, 2007). Specifically, it has been shown that superoxide overproduction as a result of the NADPH oxidase 2 (Nox2) activation leads to the loss of inhibitory capacity of PV interneurons (Schiavone et al., 2009). In our prior study, we have shown that Nox2-derived ROS in the hippocampus contributes to the development of POCD (Qiu et al., 2016). However, it remains unclear whether Nox2 activation-induced PV interneuron dysfunction is involved in the anesthesia- and surgery-induced cognitive impairment.

Therefore, we hypothesized that Nox2 activation-mediated-ROS triggering PV interneuron phenotype loss plays an important role in anesthesia- and surgery-induced cognitive impairment in aging mice. To test this hypothesis, we exposed 16-month old mice to exploratory laparotomy with isoflurane anesthesia in the presence or absence of NADPH oxidase inhibitor, apocynin (APO), to determine whether and through which mechanisms PV interneuron phenotype loss participates in the pathogenesis of POCD.

## Materials and Methods

### Animals and Ethics

**Figure 1:**
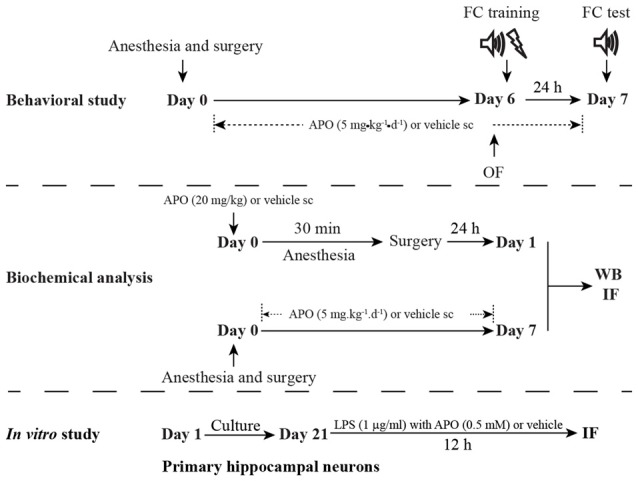
**Schematic timeline of the experimental procedure.** For the behavioral study, the mice underwent anesthesia and surgery on day 0. After that, the mice were treated with 5 mg/kg apocynin (APO) or with an equal volume of vehicle s.c. at 1 h after the surgery, and then with the same treatment once daily for the next seven consecutive days. The open field test and fear conditioning training were performed on day 6. The fear conditioning test was performed on day 7. The mice used for the biochemical analysis were not subjected to the behavioral tests and were sacrificed on days 1 and 7, respectively. The primary hippocampal neuronal culture was used to investigate the effects of oxidative stress on parvalbumin (PV) interneurons *in vitro* study.

The study protocol was approved by the Ethics Committee of Zhongda Hospital, Southeast University, and all procedures were performed in accordance with the Guidelines for the Care and Use of Laboratory Animals from the National Institutes of Health, USA. A total of 210 16-month-old male C57BL/6 mice (28–38 g) were purchased from the Animal Center of Jinling Hospital, Nanjing, China. The animals were housed in groups of 3–5 individuals per cage under standard conditions, with a 12-h light/dark cycle (light from 07:00 to 19:00) and a room temperature of 22 ± 1°C, and free access to food and water. All of the animals were given 2 weeks to acclimate to the environment before the experimentation began. The schematic timeline of the experimental procedure is summarized in Figure 1.

### Animal Model

Exploratory laparotomy was performed using aseptic procedures under isoflurane anesthesia to mimic exploratory abdominal surgery in humans. The surgical procedures were performed as described previously with minor modifications (Barrientos et al., 2012). Briefly, the mice were anesthetized by placing them in a chamber that was prefilled with 1.5% isoflurane in oxygen. The mice were exposed to isoflurane for 30 min, and then, the abdominal region was shaved and thoroughly cleaned with povidone iodine. A median abdominal incision (approximately 1-cm-long vertical incision) was made to allow penetration of the peritoneal cavity. Thereafter, the investigator inserted blunt forceps into the opening and explored the viscera, intestines and musculature. Next, sterile 4-0 chromic gut sutures were used to close the peritoneal lining and skin. The wound was dressed with polysporin (Pfizer, New York, NY, USA) to prevent infection. The animals were maintained under isoflurane anesthesia during the 10 min of the surgical procedure. The mice that served as controls received neither anesthesia nor surgery.

### APO Treatment

For the interventional study, mice were treated with an NADPH oxidase inhibitor, APO (Sigma-Aldrich, St. Louis, MO, USA). To determine the effects of APO treatment on the behaviors, the mice were treated with 5 mg/kg APO s.c. beginning at 1 h after the surgery and then once daily for the next seven consecutive days. In a separate experiment, the brain tissues were collected 7 days post-surgery for biochemical analysis. In addition, to assess the effects of the acute oxidative stress on PV expression in the brain, 20 mg/kg APO was injected s.c. just before the mice were anesthetized, and the brain tissues were collected 24 h post-surgery for biochemical analysis. The doses of APO were selected based on a previous study that suggested that single dose of 20 mg/kg APO inhibited Nox2 activation, whereas repeated administration of 5 mg/kg APO reversed sepsis-induced cognitive deficit (Hernandes et al., 2014).

### Behavioral Studies

All behavioral tests were conducted in a sound-isolated room and all behavioral data were recorded by the same two investigators, who were blinded to the animal grouping, as previously described (Ji et al., 2014; Li et al., 2014).

### Open Field

The open field test was conducted using a white opaque plastic chamber (40 cm × 40 cm × 40 cm, XR-XZ301; Shanghai Softmaze Information Technology Co., Ltd., Shanghai, China) on day 6 post-surgery to assess exploratory locomotor activity. Each mouse was gently placed in the center of the arena and left to move freely for 5 min during which the activity was automatically recorded via a video tracking system. The total distance and the time spent in the center zone of the arena (the center zone is defined as 25% of the total area) were quantified during the 5-min period.

### Fear Conditioning

Two hours after the open field test, the mice were placed in a conditioning chamber (XR-XC404; Shanghai Softmaze Information Technology Co., Ltd., Shanghai, China) and allowed to explore the chamber for 3 min before the onset of a 30-s tone (70 db, 3 kHz), followed by a 2-s footshock (0.7 mA). Then, the mice remained in the chamber for another 30 s and were then returned to their home cages. Twenty-four hours later, the mice were evaluated to quantify their fear of the conditioned context (a hippocampus-dependent task). To assess contextual fear conditioning, the mice were placed in the same chamber in which they were trained and were observed for 5 min without tone presentation or footshock during this period, the freezing behavior was scored. The auditory-cued fear test (a hippocampus-independent task) was performed 2 h later. The mice were placed in an altered chamber (i.e., a different shaped chamber, odor, no grid floor) and allowed to explore for 3 min. Then, the tone was delivered, and their freezing behavior was scored for an additional 3 min. Freezing behavior was defined as the absence of all visible movement, excluding respiration. The amount of time that the mice displayed this behavior was recorded and expressed as a percentage of the total observation period.

### Western Blot

Mice were not subjected to the behavioral tests and were instead sacrificed at the indicated time points for Western blot analysis. Hippocampal samples were homogenized in ice-cold lysis buffer (1% Nonidet P-40, 0.1% sodium deoxycholate, 0.1% SDS, 66 mM EDTA, and 10 mM Tris-HCl, pH 7.4) supplemented with a protease inhibitor cocktail, as previously described (Sadeqzadeh et al., 2011), and centrifuged at 13,000 g for 10 min at 4°C. The supernatant was saved, and the protein concentration was determined by Bradford assay. Equal amounts of sample (40 μg) were loaded per lane and electrophoresed on SDS-PAGE gels. The separated proteins were then transferred to polyvinylidene fluoride membranes. After the membranes were blocked with 5% skim milk in Tris-buffered saline with Tween (TBST), they were incubated with rabbit anti-PV (1:1000; Abcam, Cambridge, United Kingdom), goat anti-interleukin-1β (IL-1β; 1:500; R&D systems, Minneapolis, MN, USA), goat anti-p22^phox^ (1:500; Santa Cruz Biotechnology, Dallas, TX, USA), goat anti-gp91^phox^ (1:500; Santa Cruz Biotechnology), rabbit anti-Nox4 (1:1000; Abcam), rabbit anti-4-hydroxy-2-nonenal (4-HNE; 1:500; Abcam), mouse anti PGC-1α (1:500; Calbiochem, San Diego, CA, USA) and mouse anti-β-actin (1:5000; Sigma-Aldrich) overnight at 4°C. After the membranes were washed three times in TBST, they were incubated with horseradish peroxidase-conjugated secondary antibodies (goat anti-rabbit, goat anti-mouse, and rabbit anti-goat; Bioworld Technology, St. Louis Park, MN, USA), diluted to 1:5000, for 1 h at room temperature. The protein bands were detected by enhanced chemiluminescence, exposed onto X-ray film, and quantitated with Image J software (National Institutes of Health, Bethesda, MD, USA).

### Immunostaining in Slices

Mice that were not subjected to the behavioral tests were sacrificed at the indicated time points for immunofluorescence analysis. The mice were deeply anesthetized with 2% sodium pentobarbital in saline (60 mg/kg, intraperitoneally; Sigma-Aldrich) and transcardially perfused with saline and 4% paraformaldehyde in phosphate-buffered saline (PBS; pH 7.4). The brains were immediately removed, postfixed in the same 4% paraformaldehyde for 2 h and dehydrated in 30% sucrose at 4°C overnight. The brains were embedded in O.C.T. compound, cut in 10-μm-thick sections on a freezing microtome, and mounted on glass slides. The sections were blocked with 1% bovine serum albumin (BSA) for 1 h at room temperature and then incubated with primary antibodies, including rabbit anti-PV (1:600; Abcam), mouse anti-PV (1:600; Chemicon, Temecula, CA, USA), goat anti-gp91^phox^ (1:200; Santa Cruz Biotechnology), goat anti-p22^phox^ (1:200; Santa Cruz Biotechnology), mouse anti-8-hydroxy-2’-deoxyguanosine (8-OH-dG; 1:200; Santa Cruz Biotechnology) and rabbit anti-4-HNE (1:200; Abcam), in 1% BSA at 4°C overnight. After the sections were washed three times with PBS, they were incubated with appropriate secondary antibodies, including goat anti-rabbit IgG-FITC or -Cy3 (1:300; Santa Cruz Biotechnology), goat anti-mouse IgG-FITC or -Cy3 (1:600; Bioworld Technology) and donkey anti-goat IgG-Cy3 (1:800; Abcam), for 1 h at room temperature. After the secondary antibody was washed out, the sections were incubated with 4′,6-diamidino-2-phenyl-indole (DAPI) for nuclear staining. Fluorescence images were captured at 40× magnification using a confocal microscope (Leica, TCS SP2, Germany). We randomly selected six slices per animal across the hippocampal region between Bregma −1.70 and −2.30. The fluorescence intensity and the number of PV interneurons were quantified by the NIH Image J. The mean fluorescence/cell was calculated and normalized in the control group and expressed as 1.

### NADPH Oxidase Activity

NADPH oxidase activity was evaluated using an assay kit (Genmed Scientifics Inc., Wilmington, DE, USA) according to the manufacturer’s instructions (Zhang et al., 2014). Briefly, the hippocampus was homogenized in PBS and centrifuged at 2500× g for 10 min. The supernatant was collected and incubated with NADPH. NADPH oxidase activity was assessed by monitoring the rate of NADPH consumption, which was inhibited by diphenyliodonium and measured by spectrophotometry at 340 nm.

### Neuronal Cultures

Primary hippocampal neurons were cultured as previously described with minor modifications (Beaudoin et al., 2012). The brains from postnatal (P0-P1) C57BL/6 mice were removed quickly, and the hippocampus was dissected and mechanically disaggregated by gentle trituration using a Pasteur pipette. Dissociated cells were cultured in neurobasal medium supplemented with 1× B27, 600 μM L-glutamine, and 1× penicillin/streptomycin (Life Technologies, Grand Island, NY, USA). The cells were seeded on polylysine-coated glass coverslips at a density of ~1.4 × 10^4^ cells per cm^2^ in 15-mm multiwell plates. Every 3 days, half of the medium was replaced with freshly prepared medium. After 21 days of culturing, the neurons became aged and were exposed to Lipopolysaccharide (LPS, 1 μg/ml, Sigma-Aldrich), which was reported as a stressor to induce oxidative/inflammatory stress signaling (Joseph et al., 2010), or vehicle in the absence or presence of 0.5 mM APO (Behrens et al., 2007). After 12 h, the cultured neurons were fixed as previously described and then immunostained (Beaudoin et al., 2012).

### Immunostaining in Dissociated Neurons

Dissociated neurons were fixed with a 4% paraformaldehyde/4% sucrose mixture in PBS that was warmed to 37°C in advance for 10 min at room temperature. The neurons were then permeabilized by incubation with a 0.1% (vol/vol) Triton X-100 solution in PBS for 10 min at room temperature and washed gently with PBS. The neurons were blocked with 1% BSA in PBS for 1 h at room temperature and incubated at 4°C overnight with primary antibodies, including rabbit anti-PV (1:600; Abcam), goat anti-gp91^phox^ (1:200; Santa Cruz Biotechnology), goat anti-p22^phox^ (1:200; Santa Cruz Biotechnology), and mouse anti-PSD95 (1:200; Chemicon), diluted in 1% BSA. After the neurons were washed three times with PBS, they were incubated with secondary antibodies, including goat anti-rabbit IgG-FITC (1:300; Santa Cruz Biotechnology), goat anti-mouse IgG-Cy3 (1:600; Bioworld Technology) and donkey anti-goat IgG-Cy3 (1:800; Abcam), for 1 h at room temperature. After the neurons were washed three times with PBS, they were incubated with DAPI to label nuclei. Fluorescence images were obtained by confocal scanning microscopy (Leica, TCS SP2, Germany). For a given sample, 5–10 images, which were taken at 1-μm intervals, were collapsed to generate a projected image. The number of PSD95-positive puncta was measured by Image J (National Institutes of Health, Bethesda, MD, USA).

### Statistical Analysis

Statistical analyses were performed using the Statistical Package for Social Sciences (SPSS; version 16.0, Chicago, IL, USA). The data are expressed as the mean ± S.E.M. Differences between two groups were determined using an independent *t* test. The main effects of surgery and APO treatment and the interaction between them were evaluated by a 2 × 2 factorial analysis of variance (ANOVA) model that included assessment of the interaction. When an interaction effect was identified, one-way ANOVA followed by Tukey’s test was applied. For data that were not normally distributed, a non-parametric Kruskal Wallis test followed by Dunn’s test was applied. Bivariate relationships were evaluated using Pearson correlation coefficients. A *p* < 0.05 was regarded as statistically significant.

## Results

### Surgery with Anesthesia Induced Cognitive Impairment, Up-Regulation of Nox2 and Down-Regulation of PV in the Hippocampus of Aging Mice

**Figure 2:**
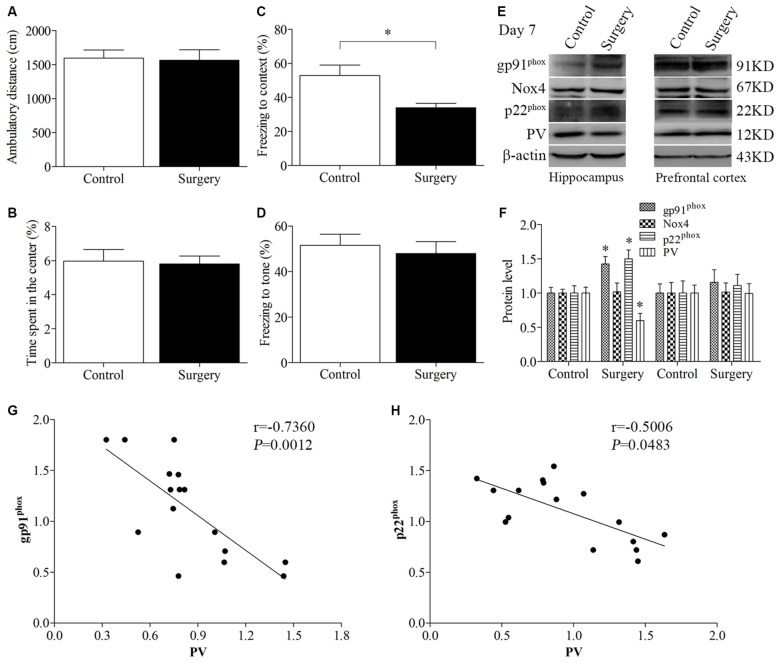
**Impaired contextual fear conditioning behavioral phenotype, up-regulated NADPH oxidase 2 (Nox2), and down-regulated PV in the hippocampus on day 7 post-surgery.** There was no significant difference in **(A)** the ambulatory distance and **(B)** time spent in the center in the open field test between the two groups on day 6 post-surgery. **(C)** The freezing time to context was decreased in the surgery group compared with the control group. **(D)** No significant difference was detected in the cued fear conditioning between the two groups. **(E,F)** Western blot results demonstrated that the expression of PV was decreased in the hippocampus but not in the prefrontal cortex in the surgery group compared with the control group. The expression of gp91^phox^ and p22^phox^ was increased in the hippocampus but not in the prefrontal cortex in the surgery group. There was no significant difference in the expression of Nox4 in the hippocampus or prefrontal cortex between the two groups. Correlation analysis showed that the expression of PV was negatively correlated with the **(G)** gp91^phox^ and **(H)** p22^phox^ expression in the hippocampus. The data are presented as the mean ± S.E.M. (10 mice in each group in **A–D**, 5 mice in each group in **E–H**). *p < 0.05 compared with the control group.

The open field test was conducted to investigate whether surgery with anesthesia influence locomotor activity and exploratory behavior. During the 5-min test period, the total distance (*p* = 0.870, Figure 2A) and the time spent in the center of the arena (*p* = 0.851, Figure 2B) had no significant difference between the control and surgery groups. Next, we used the fear conditioning paradigm to assess memory performance. Twenty-four hours after the training session, the mice in the surgery group had less freezing time in the contextual fear conditioning test than the control mice (*p* = 0.014, Figure 2C); however, there was no significant difference in post-tone freezing time in the auditory-cued fear test between the two groups (*p* = 0.619 Figure 2D), suggesting that anesthesia and surgery could impair hippocampus-dependent memory.

The expression of PV was decreased in the hippocampus (*p* = 0.018, Figures 2E,F) but not in the prefrontal cortex (*p* = 0.970, Figures 2E,F) in the surgery group compared with the control group on day 7 post-surgery. To determine the effect of surgery on NADPH oxidase, we detected the expression of its subunits, gp91^phox^ (Nox2), Nox4 and p22^phox^. A significant increase in gp91^phox^ and p22^phox^ expression was observed in the hippocampus but not the prefrontal cortex (hippocampus: all *p* < 0.05; cortex: all *p* > 0.05; Figures 2E,F). In addition, there was no significant difference in the expression of Nox4 in the hippocampus and prefrontal cortex between the two groups (*p* > 0.05), suggesting that surgery selectively influenced Nox2. Since the protein levels in the prefrontal cortex were not different between the two groups, we only performed biochemical analysis in the hippocampus in our subsequent experiments.

Furthermore, the expression of PV was negatively correlated with the gp91^phox^ (Pearson correlation coefficients, *r* = −0.7360, *p* = 0.0012, Figure 2G) and p22^phox^ (Pearson correlation coefficients, *r* = −0.5006, *p* = 0.0483, Figure 2H) expression in the hippocampus.

**Figure 3:**
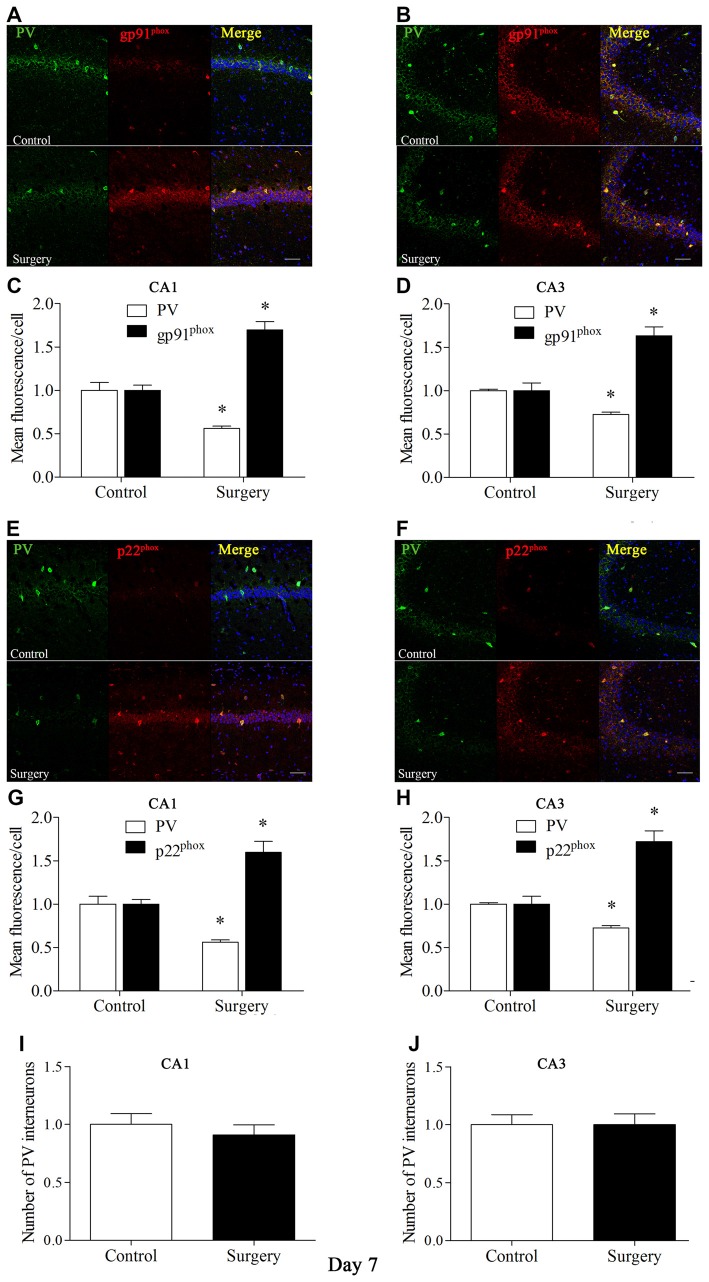
**Immunofluorescent co-localization of PV and gp91^phox^ or p22^phox^ in areas Carbonic anhydrase 1 (CA1) and CA3 of the hippocampus on day 7 post-surgery. (A,B)** Representative images of PV (green) and gp91^phox^ (red) expression in areas CA1 and CA3. **(C,D)** Quantification of PV and gp91^phox^ fluorescence in areas CA1 and CA3. **(E,F)** Representative images of PV (green) and p22^phox^ (red) expression in areas CA1 and CA3. **(G,H)** Quantification of PV and p22^phox^ fluorescence in areas CA1 and CA3. **(I,J)** Analysis of number of PV interneurons in areas CA1 and CA3. Scale bar = 50 μm. 6-diamidino-2-phenyl-indole (DAPI) staining is shown in blue. The data are presented as the mean ± S.E.M. (8 mice in each group). *p < 0.05 compared with the control group.

To further test the hypothesis that Nox2 activation in PV interneurons contributes to PV interneuron phenotype loss, we performed *in vivo* double-immunofluorescence labeling for PV and gp91^phox^ as well as for PV and p22^phox^. As revealed in Figure 3, gp91^phox^ and p22^phox^ immunoreactivity mainly colocalized to PV interneurons. In contrast to the decreased PV immunoreactivity on day 7 post-surgery, gp91^phox^ and p22^phox^ immunoreactivity was increased in the hippocampus on day 7 post-surgery in the surgery mice compared with the control mice (all *p* < 0.01, Figures 3A–H), which was consistent with the Western blot results (Figures 2E,F). We also analyzed the number of PV interneurons in areas CA1 and CA3 of the hippocampus on day 7 post-surgery and the result showed no significance between the control and surgery groups (all *p* > 0.05, Figures 3I,J).

### APO Treatment Prevented Surgery-Induced Cognitive Impairment, Decreased PV Expression, and Increased Inflammatory Responses and Oxidative Stress in the Hippocampus, at Least Partially, by Inhibiting NADPH Oxidase Activity

**Figure 4:**
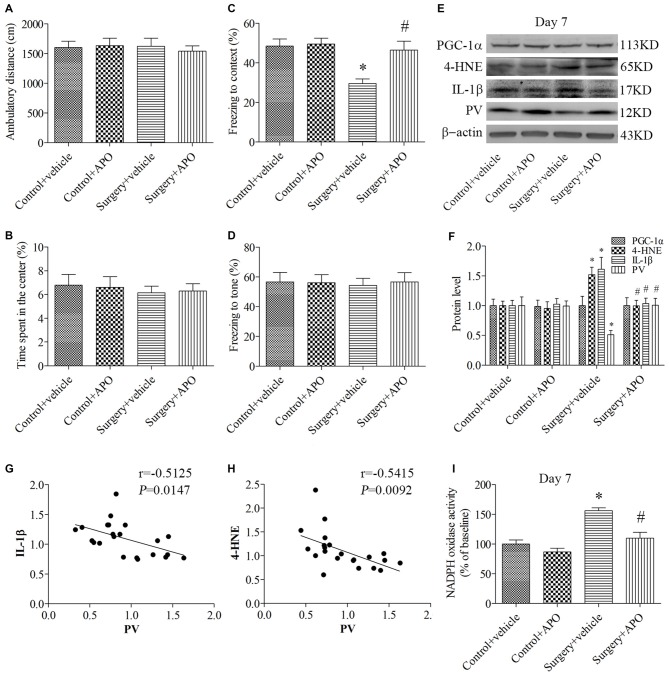
**Rescuing effects of APO on surgery-induced impaired cognition, decreased PV expression, increased interleukin-1β (IL-1β) and 4-HNE expression and nicotinamide adenine dinucleotide phosphate (NADPH) oxidase activity in the hippocampus on day 7 post-surgery.** There was no significant difference in **(A)** the ambulatory distance and **(B)** time spent in the center in the open field test among the four groups on day 6 post-surgery. **(C)** The freezing time to context was decreased in the surgery + vehicle group compared with the control + vehicle group, which was rescued by APO treatment. **(D)** No significant difference was detected in the cued fear conditioning among the four groups. **(E,F)** Western blot results demonstrated the decreased PV expression and increased IL-1β and 4-HNE expression in the hippocampus in the surgery + vehicle group compared with the control + vehicle group, which was rescued by APO treatment. There was no significant difference in the expression of proliferator-activated receptor coactivator 1α (PGC-1α) among the four groups. Correlation analysis showed that the PV expression was negatively correlated with the **(G)** IL-1β and **(H)** 4-HNE expression. **(I)** NADPH oxidase activity was increased in the surgery + vehicle group compared with the control + vehicle group, which was rescued by APO treatment. The data are presented as the mean ± S.E.M. (10 mice in each group in **A–D**, 5 mice in each group in **E–I**). *p < 0.05 compared with the control + vehicle group, ^#^*p* < 0.05 compared with the surgery + vehicle group.

In the open field test, when the ambulatory distance (Figure 4A) and the time spent in the center (Figure 4B) were examined by 2 × 2 ANOVA, there was no interaction between surgery and APO treatment (all *p* > 0.05). In the contextual fear conditioning test, the freezing time to context was decreased in the surgery + vehicle group compared with the control + vehicle group, which was rescued by APO treatment (*p* = 0.008, Figure 4C). There was no interaction between surgery and APO treatment on post-tone freezing times in any of the four groups (*p* > 0.05, Figure 4D). These results suggested that the behavioral difference observed in the fear conditioning test could not be attributed to lethargy or any other nonspecific effect of anesthesia and surgery.

The expression of PV, IL-1β and 4-HNE (a byproduct of lipid peroxidation) was analyzed by 2 × 2 ANOVA, which indicated that the main effects of surgery and APO treatment as well as the surgery × APO treatment interaction effect were all significant (all *p* < 0.05). The expression of IL-1β and 4-HNE was up-regulated, but the expression of PV was down-regulated after surgery (all *p* < 0.05, Figures 4E,F), which were rescued by APO treatment (all *p* < 0.05, Figures 4E,F). Interestingly, the expression of the transcriptional coactivator peroxisome proliferator-activated receptor coactivator 1α (PGC-1α), a master regulator of metabolism, was comparable among the four groups (*p* > 0.05, Figures 4E,F). Linear regression analysis revealed that the expression of PV was negatively correlated with the IL-1β (Pearson correlation coefficients, *r* = −0.5125, *p* = 0.0147, Figure 4G) and 4-HNE (Pearson correlation coefficients, *r* = −0.5415, *p* = 0.0092, Figure 4H) expression in the hippocampus. We further determined NADPH oxidase activity in the hippocampus and showed NADPH oxidase activity was increased following surgery (*p* = 0.001, Figure 4I), whereas APO treatment inhibited this increase in NADPH oxidase activity (*p* = 0.004, Figure 4I).

### Surgery-Induced Up-Regulation of Markers of Oxidative Stress in Hippocampal PV Interneurons was Attenuated by APO Treatment

**Figure 5:**
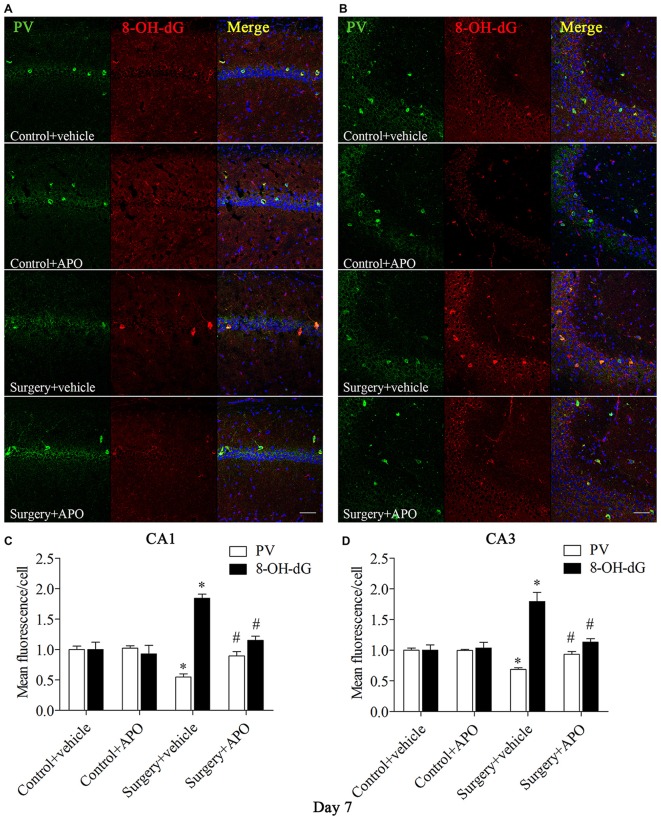
**Immunofluorescent co-localization of PV and 8-OH-dG in areas Carbonic anhydrase 1 (CA1) and CA3 of the hippocampus on day 7 post-surgery. (A,B)** Representative images of PV (green) and 8-OH-dG (red) expression in areas CA1 and CA3. **(C,D**) Quantification of PV and 8-OH-dG fluorescence in areas CA1 and CA3. Scale bar = 50 μm. DAPI staining is shown in blue. The data are presented as the mean ± S.E.M. (8 mice in each group). *p < 0.05 compared to the control + vehicle group, ^#^*p* < 0.05 compared to the surgery + vehicle group.

**Figure 6:**
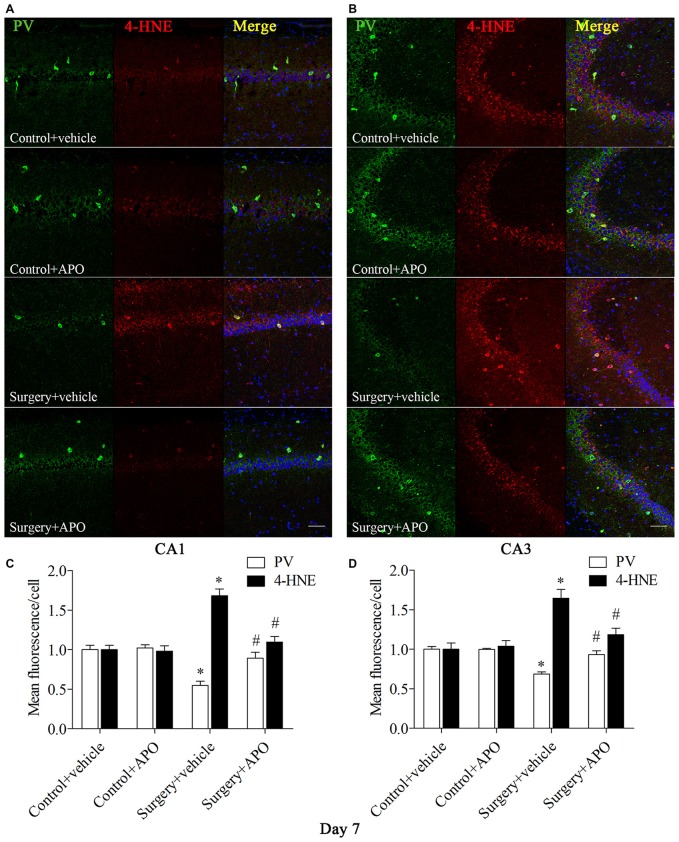
**Immunofluorescent co-localization of PV and 4-HNE in areas CA1 and CA3 of the hippocampus on day 7 post-surgery. (A,B**) Representative images of PV (green) and 4-HNE (red) expression in areas CA1 and CA3. **(C,D)** Quantification of PV and 4-HNE fluorescence in areas CA1 and CA3. Scale bar = 50 μm. DAPI staining is shown in blue. The data are presented as the mean ± S.E.M. (8 mice in each group). *p < 0.05 compared to the control + vehicle group, ^#^*p* < 0.05 compared to the surgery + vehicle group.

We used double-immunofluorescence to detect the colocalization of markers of oxidative stress and PV in the hippocampus. Oxidative stress was examined by 8-OH-dG (a marker of DNA oxidative damage) and 4-HNE. The 8-OH-dG and 4-HNE immunoreactivity was mainly colocalized to PV interneurons. Most PV-positive cells in areas CA1 and CA3 were also immunoreactive for 8-OH-dG (all *p* < 0.001, Figure 5) and 4-HNE (all *p* < 0.001, Figure 6) in the surgery + vehicle group on day 7 post-surgery, suggesting that PV interneurons are vulnerable to oxidative stress. Notably, APO treatment could attenuate surgery-induced oxidative stress in PV interneurons (all *p* < 0.01, Figures 5, 6).

### A Single Large Dose of APO Pretreatment Also Attenuated Surgery-Induced Acute Oxidative Stress, Inflammatory Response and Down-Regulation of PV in the Hippocampus

**Figure 7:**
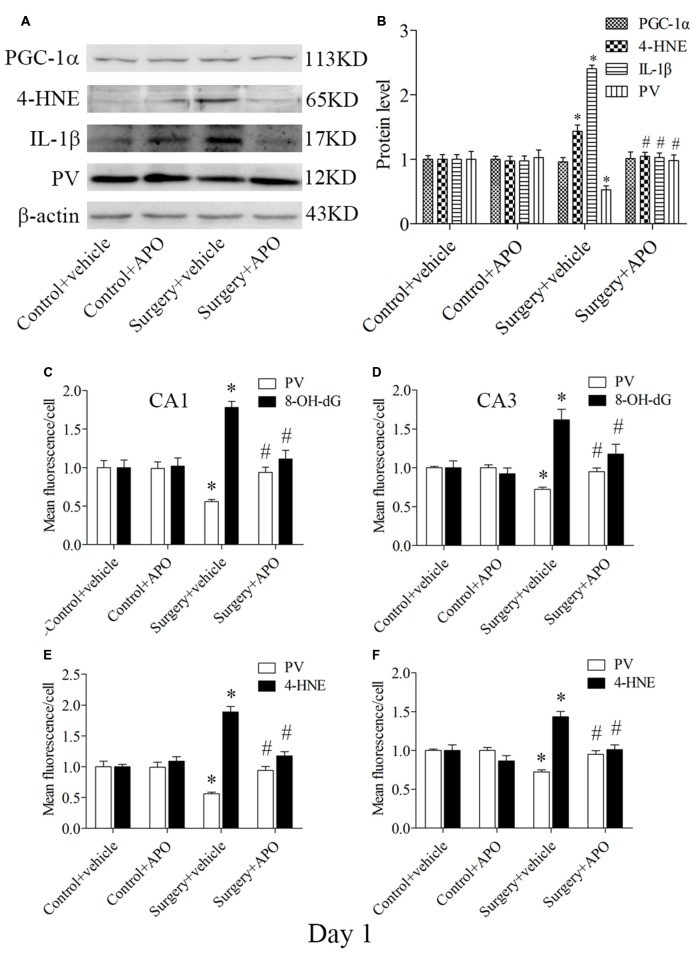
**Hippocampal oxidative stress detected by Western blot and double-immunofluorescence staining in PV interneurons on day 1 post-surgery. (A)** Representative Western blot images of PV, IL-1β, 4-HNE and PGC-1α in the hippocampus in the four groups; **(B)** Quantitative analysis. **(C,D)** Quantification of PV and 8-OH-dG fluorescence in areas CA1 and CA3. **(E,F)** Quantification of PV and 4-HNE fluorescence in areas CA1 and CA3. The data are presented as the mean ± S.E.M. (5 mice in each group in **A,B**, 8 mice in each group in **C–F**). *p < 0.05 compared to the control + vehicle group, ^#^*p* < 0.05 compared to the surgery + vehicle group.

Western blot results for PV, IL-1β and 4-HNE were evaluated by 2 × 2 ANOVA, which revealed significant main effects of surgery and APO treatment as well as an interaction between surgery and APO treatment (all *p* < 0.05, Figures 7A,B). PV expression was up-regulated, and IL-1β and 4-HNE expression was down-regulated in the surgery + APO group compared with the surgery + vehicle group (all *p* < 0.05, Figure 7B). There was no significance in PGC-1α expression among the four groups (*p* > 0.05, Figure 7B). The results of the double-immunofluorescence analysis of the colocalization of PV with 8-OH-dG and 4-HNE in the hippocampus on day 1 post-surgery were consistent with those on day 7 post-surgery (immunofluorescence images not shown, all *p* < 0.05, Figures 7C–F).

### LPS Exposure Increased Nox2 Expression and Decreased PV Immunoreactivity in Primary Hippocampal Neuronal Cultures

**Figure 8:**
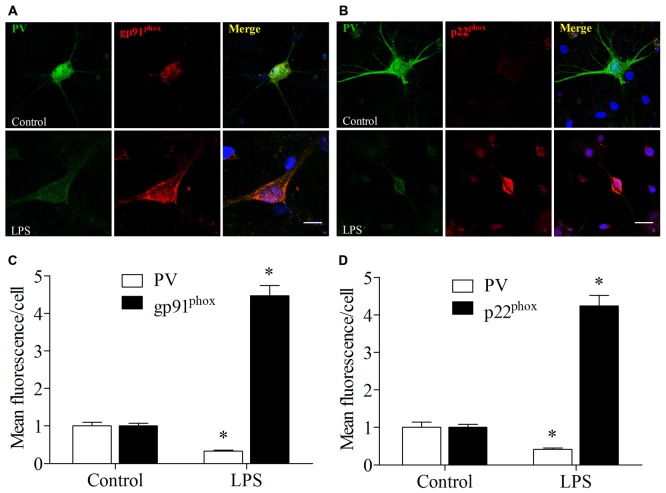
**Lipopolysaccharide (LPS) exposure increased gp91^phox^ and p22^phox^ expression and decreased PV immunoreactivity in primary neuronal cultures. (A,B)** Representative images of PV (green) and gp91^phox^ (red) or p22^phox^ (red) expression in primary hippocampal neuronal cultures. **(C,D)** Quantification of PV, gp91^phox^, and p22^phox^ fluorescence in the primary hippocampal neuronal cultures. Scale bar = 20 μm. DAPI staining is shown in blue. The data are presented as the mean ± S.E.M. (12 cells in each group). *p < 0.05 compared to the control group.

These *in vitro* experiments were used as a complementary means of investigating the mechanisms underlying the oxidative stress-induced down-regulation of PV. We used double-immunofluorescence to detect colocalization of gp91^phox^ and p22^phox^ with PV in the cultured hippocampal neurons. Increases in gp91^phox^ and p22^phox^ immunoreactivity and decrease in PV immunoreactivity were observed in the neurons after 12 h exposure to LPS (all *p* < 0.001, Figure 8).

### LPS Exposure-Induced Decrease in the Number of Excitatory Synapses onto PV Interneurons Was Ameliorated by APO Treatment in Primary Hippocampal Neuronal Cultures

**Figure 9:**
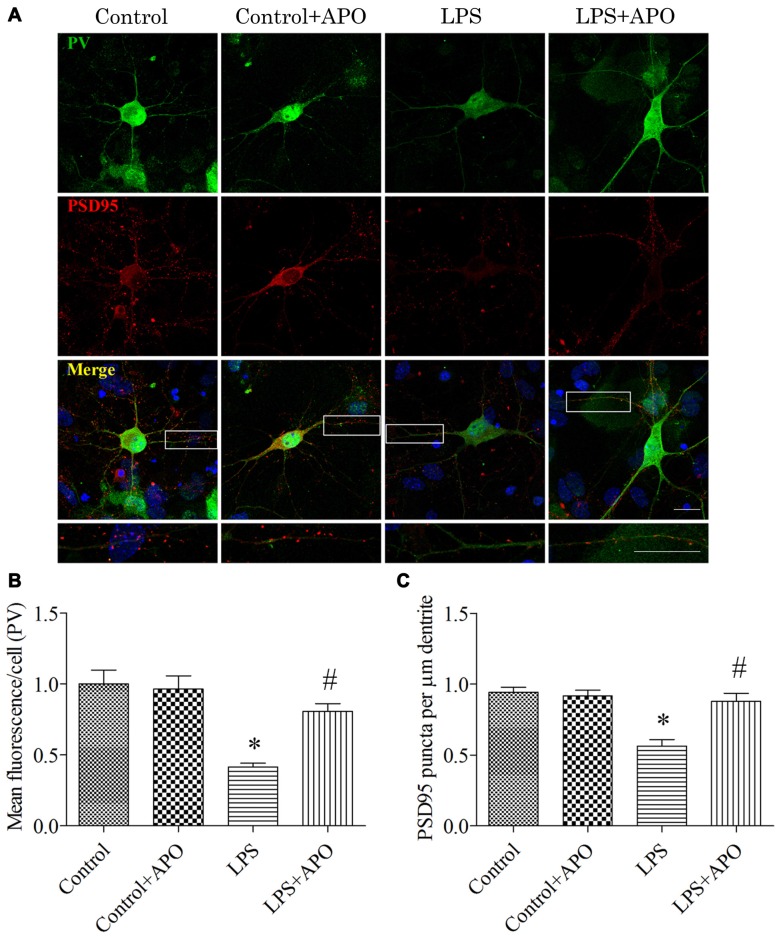
**Rescuing effects of APO on LPS-stimulated decreases in the PV expression and number of excitatory synapses onto PV interneurons in primary neuronal cultures. (A)** Cultured hippocampal neurons stained for PV (green) and PSD95 (red); the boxed area is enlarged in the bottom image. **(B)** Quantification of PV fluorescence in the primary hippocampal neuronal cultures. **(C)** The number of PSD95-positive puncta per μm dendrite in the four groups. Scale bar = 20 μm. DAPI staining is shown in blue. The data are presented as the mean ± S.E.M. (12 cells in each group). *p < 0.05 compared to the control group, ^#^*p* < 0.05 compared to the LPS group.

To determine whether the PV interneuron phenotype loss was attributed to oxidative stress, we quantified the number of excitatory synapses onto PV interneurons after 12 h exposure to LPS. Excitatory synapses were identified by staining for PSD95, a major scaffolding protein that is localized to excitatory synapses. To avoid the bias in data collection, puncta were counted within dentritic segments of the same length. As shown in Figure 9, 2 × 2 ANOVA analysis of the immunoreactivity of PV and the number of PSD95-positive puncta onto PV interneurons revealed significant main effects of surgery and APO treatment as well as an interaction between surgery and APO treatment (all *p* < 0.05). PV immunoreactivity and the number of PSD95-positive puncta onto PV interneurons were decreased after exposure to LPS (all *p* < 0.001, Figure 9), which were ameliorated by APO treatment (all *p* < 0.001, Figure 9).

## Discussion

Our results showed that Nox2-mediated ROS played a key role in phenotype loss of hippocampal PV interneurons and consequently led to cognitive impairment in aging mice after laparotomy surgery with isoflurane anesthesia. Notably, treatment with APO, an NADPH oxidase inhibitor, abolished the enhanced oxidative stress and neuroinflammation and subsequently prevented the dysfunction of PV interneurons in the hippocampus, which ultimately improved postoperative cognitive impairment in mice.

POCD can affect a wide variety of cognitive domains, such as attention, memory, executive function and the speed of information processing (Hovens et al., 2012). The incidence of POCD varies widely, from 41–75% at 7 days to 18–45% at 3 months postoperatively across different studies. POCD is also associated with prolonged hospitalization, reduced quality of life, and increased morbidity and mortality (Deo et al., 2011). Although the precise mechanisms remain unclear, accumulating evidence suggests that neuroinflammation plays a central role in surgery-induced cognitive impairment (Cibelli et al., 2010; Barrientos et al., 2012; Zhang et al., 2014; Lu et al., 2015), which was confirmed by the up-regulation of IL-1β in the hippocampus in the present study. Recently, oxidative stress has been proposed to be involved in the development of POCD in rats (An et al., 2013; Yuan et al., 2014). Overproduction of ROS can cause oxidative damage of lipids, proteins and DNA. Our previous study has demonstrated that oxidative stress in the brain, especially in the hippocampus played a key role in the pathogenesis of POCD (Qiu et al., 2016). In the present study, we also revealed that the surgery resulted in enhanced oxidative stress in the hippocampus, as indicated by higher levels of 8-OH-dG and 4-HNE. Since the poor knowledge about the role of oxidative stress in POCD, we further explore the mechanisms whereby the oxidative stress be involved in the pathogenesis of POCD in the present study.

PV interneurons, a subset of inhibitory neurons, are involved in the generation of gamma oscillations and play an important role in cognitive performance. For example, an early life stress such as maternal separation can lead to reduced PV expression in the rat prefrontal cortex as well as work memory deficit in adolescence (Brenhouse and Andersen, 2011). A down-regulation of PV in the medial prefrontal cortex followed by a reduction in coordinated neural activity and behavioral impairment has been shown in a rodent model of schizophrenia (Lodge et al., 2009). We previously revealed that a decreased hippocampal PV expression following with a disruption of neuregulin 1-ErbB4 signaling contributed to isoflurane-induced hippocampus-dependent cognitive impairment in aging mice (Li et al., 2014). Consistently, we found in the present study that PV interneuron phenotype loss was only observed in the hippocampus and co-occurs with the hippocampus-dependent cognitive impairment after the surgery with isoflurane anesthesia. We therefore explore the mechanisms underlying the involvement of the hippocampal PV interneurons in the development of POCD and the association of the PV interneurons with the oxidative stress.

Accumulating evidence indicates that PV interneurons are susceptible to oxidative stress in response to adverse stimulation. One reason for this susceptibility might be that these cells require considerable energy due to their fast-spiking properties (Steullet et al., 2010). The NADPH oxidases are well known generators of superoxide. Nox2 and Nox4 are the main subunits expressed in the central nervous system (Infanger et al., 2006). Gp91^phox^, also termed Nox2, requires the membrane protein p22^phox^, as well as a series of cytosolic proteins that are involved in its activation. The activity of Nox4 also depends on p22^phox^, but p22^phox^ seems to be a constitutive enzyme that does not require cytosolic components for activation. In the present study, the increases in the Nox2 expression and NADPH oxidase activity, but not Nox4 expression, were observed in the hippocampus on day 7 post-surgery, suggesting that Nox2 in the hippocampus is the main oxidase isoform affected by anesthesia and surgery. It was reported that Nox2-derived oxidative stress was involved in the phenotype loss of PV interneurons and the development of behavioral alteration after social isolation (Schiavone et al., 2009). In line with this notion, our previous study in a POCD model demonstrated that the activation of Nox2 in the hippocampus was attributed to the cognitive impairment after anesthesia and surgery (Qiu et al., 2016). Furthermore, we found in the present study that PV interneuron phenotype loss was only observed in the hippocampus but not in the prefrontal cortex on day 7 post-surgery with isoflurane anesthesia. Therefore, these results support our hypothesis that Nox2 activation-mediated-ROS triggers the phenotype loss of PV interneurons in the hippocampus and results in consequent cognitive impairment after anesthesia and surgery in aging mice.

NADPH oxidase-dependent production of superoxide is involved in the increase in oxidative stress that is observed in a variety of brain disorders, including cerebral ischemia (Sugawara and Chan, 2003; Suh et al., 2008; Yoshioka et al., 2011), traumatic brain injury (Dohi et al., 2010), sepsis-associated encephalopathy (Hernandes et al., 2014), and psychiatric disorders as well as Alzheimer’s disease, Parkinson’s disease and other neurodegenerative diseases (Sorce and Krause, 2009). To further identify whether this phenotype loss in PV interneurons was associated with Nox2 activation, we performed immunofluorescence to detect the colocalization of gp91^phox^ and p22^phox^ with PV in the hippocampus *in vivo* and *in vitro*. We showed that PV immunoreactivity was reduced, but gp91^phox^ and p22^phox^ immunoreactivity was increased in areas CA1 and CA3 of the hippocampus following surgery. The *in vitro* study also confirmed that the PV interneuron phenotype loss in the hippocampus was associated with increased Nox2 expression. Conversely, PGC-1α, a primary regulator of metabolism, reportedly plays a central role in neuroprotection through the activation of genes that are involved in ROS metabolism and is required for proper PV expression in multiple tissues (Lucas et al., 2010). In our study, we found that surgery did not influence the level of PGC-1α in the hippocampus, suggesting that mitochondria-derived ROS minimally affect the PV interneuron phenotype loss. Based on these results, we proposed that this PV interneuron phenotype loss in the hippocampus is most likely due to the Nox2 activation specifically in hippocampal PV interneurons, although our data could not exclude the influence of ROS from other cell types or regions.

The balance between excitation and inhibition is important for the homeostatic control of normal brain function and behavior (Ting et al., 2011). The excitability of neurons relies on the summation of excitatory and inhibitory signals, which, in turn, is regulated by the number of excitatory and inhibitory synapses they receive (Ting et al., 2011; Donato et al., 2013). To investigate the mechanism underlying oxidative stress induced-dysfunction of PV interneurons, we examined the level of PSD95, a major scaffolding protein that is localized onto excitatory synapses. Our recent study has demonstrated that LPS-stimulation can lead to a decrease of PSD95-positive puncta onto PV interneurons in primary hippocampal neurons cultured for 14 days (Ji et al., 2015). We here cultured the hippocampal neurons for a longer time (21 days) with a shorter LPS exposure time (12 h), which might be more specific for aging mice following surgery. We observed that LPS-stimulation induced a decrease in the number of PSD95-positive puncta onto PV interneurons, which was attenuated by APO treatment. These results were supported by one previous study demonstrating that impaired excitatory synapse maturation in GABAergic interneurons is associated with neuropsychiatric disorders (Yin et al., 2013).

There are some limitations in the study design. First, using the PV-Nox2/gp91phox^−/−^ mice can provide more specific evidence regarding whether the inhibition of Nox2 in PV interneurons can influence the PV phenotype in the hippocampus than using APO. Since we could not obtain the PV-Nox2/gp91phox^−/−^ mice currently, this genetic approach will be performed as the next step to build on the findings of the present study. Second, it is better to have a control group that treated only with isoflurane. However, it has been previously demonstrated that anesthesia alone for a period comparable to the duration of surgery does not induce cognitive impairment in animal models (Cibelli et al., 2010; Hovens et al., 2014). Also, it is uncommon that patients receive anesthesia alone but not surgery in clinical practice. For these reasons, we did not include the isoflurane alone group. Third, in order to minimize the animals used, we only detected the PV expression within 7 days after the surgery, during which a high occurrence of POCD was reported (Wan et al., 2007; Cao et al., 2010; Kamer et al., 2012), but did not analyze how the expression of PV changed with the development of POCD. Finally, even though the exposure of primary hippocampal neurons to LPS can induce oxidative stress, it is not directly related to the *in vivo* POCD model. Therefore, the *in vitro* model with LPS is not the best model as a complimentary means to study the underlying mechanisms.

In summary, our study demonstrated that Nox2-derived ROS production, at least in part, plays a central role in anesthesia- and surgery-induced hippocampal PV interneuron phenotype loss, diminished cortical inhibitory drive and consequent cognitive impairment. Hence, identifying viable therapeutic strategies to tackle oxidative stress and the consequent PV interneuron disturbance may provide an exciting therapeutic strategy for POCD.

## Author Contributions

J-JY, M-HJ, L-LQ designed the project. L-LQ, DL, HZ, YSS, Y-JL, DW and JC performed the experiments. J-JY, L-LQ and M-HJ analyzed the data and wrote the article. J-JY and YSS secured the funds to support this project. All the authors read and approved the manuscript.

## Conflict of Interest Statement

The authors declare that the research was conducted in the absence of any commercial or financial relationships that could be construed as a potential conflict of interest.
